# High-attenuation mucus and acute atelectasis in allergic bronchopulmonary aspergillosis

**DOI:** 10.1093/qjmed/hcae244

**Published:** 2024-12-26

**Authors:** Jianmin Duan, Xi Yu, Hong Zheng

**Affiliations:** Department of Respiratory and Critical Care Medicine, Tianjin First Central Hospital, Tianjin, China; Department of Respiratory and Critical Care Medicine, Tianjin First Central Hospital, Tianjin, China; Department of Respiratory and Critical Care Medicine, Tianjin First Central Hospital, Tianjin, China

**Figure 1.**
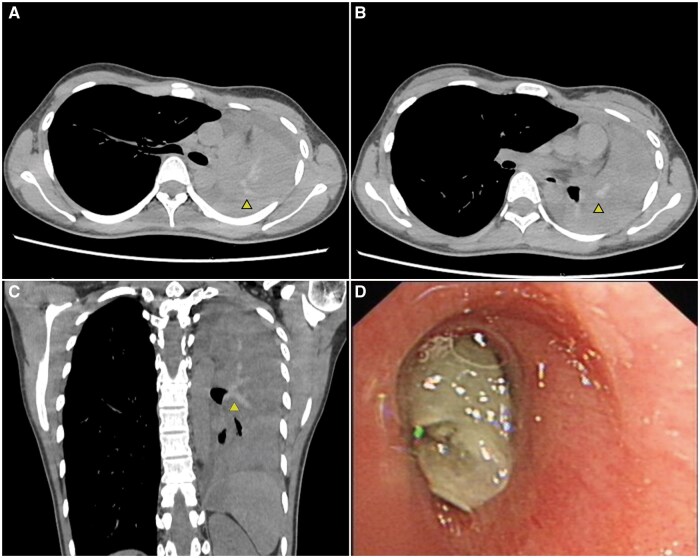
Computed tomography (CT) findings: axial view (**A**, **B**) and coronal view (**C**) showing high-attenuation mucus (arrows) within the left main bronchus and its branches, extending along the tracheal direction, radiating from the left hilum to the periphery with complete atelectasis of the left lung. (**D**) Flexible bronchoscopy showing a gelatinous mucus plug completely obstructed the left main bronchus.

A 21-year-old female college student presented with a 3-year history of undiagnosed and untreated seasonal wheezing in autumn. She developed a cough 5 days prior, which progressively worsened. She was admitted to the emergency department due to acute dyspnea. Physical examination showed absent breath sounds on the left side, with dullness to percussion, and coarse breath sounds on the right. A chest computed tomography (CT) scan showed collapse of the left hemithorax, complete atelectasis of the left lung, minimal left pleural effusion and a shift of the mediastinum to the left. On mediastinal window settings, a high-attenuation mucus (HAM) sign was observed within the left main bronchus and its branches, extending along the tracheal direction, with a CT attenuation value of approximately 105 HU and bronchoscopic examination revealed a complete obstruction of the left main bronchus by a gelatinous mucus plug (Figure 1), which can be removed by a cryoprobe. Pathological examination revealed abundant Aspergillus species within the mucus plug. The patient’s blood eosinophil count was 2.1 × 10^9^/l, serum IgE level was >1000 IU/ml, and Aspergillus fumigatus-specific IgE was 75.3 kUA/l. Based on the medical history, clinical tests and imaging data, the patient was diagnosed with allergic bronchopulmonary aspergillosis (ABPA) complicated by a complete mucus plug obstruction of the left airway. Treatment with corticosteroids and voriconazole led to improvement and full re-expansion of the left lung.

ABPA is an allergic reaction to inhaled Aspergillus species, often presenting with uncontrollable asthma.^1^ The acute atelectasis as an initial presentation, as seen in this case, is relatively rare. The accumulation of secretions or mucus plugs in the airways of ABPA patients can lead to mucous impaction. More than 20% of ABPA cases exhibit HAM, characterized on CT by tubular or branching high-density shadows extending from the hilum to the periphery, forming a ‘glove finger sign’, with CT values exceeding 70 HU.^2^ This is associated with increased mucus viscosity due to Aspergillus hyphae and deposition of calcium and other metal ions (iron and manganese).^3^ HAM is identified as a distinctive and diagnostically relevant imaging hallmark of ABPA.
